# Disruption of a selective vesicle pool upon retrograde amnesia dissociates memory at presynaptic terminals

**DOI:** 10.1073/pnas.2514875123

**Published:** 2026-03-05

**Authors:** Shun Hiramatsu, Kaito Kabetani, Shu Kondo, Hiromu Tanimoto

**Affiliations:** ^a^Department of Integrative Life Sciences, Graduate School of Life Sciences, Tohoku University, Sendai 980-8577, Japan; ^b^Department of Biological Science and Technology, Faculty of Advanced Engineering, Tokyo University of Science, Tokyo 125-8585, Japan

**Keywords:** retrograde amnesia, synaptic vesicles, *Drosophila*, memory consolidation

## Abstract

A single learning episode induces both labile and consolidated forms of aversive olfactory memory in *Drosophila melanogaster*. Retrograde amnesia triggered by post-learning perturbations specifically impairs the labile memory. However, synaptic mechanisms for this selectivity remain elusive. Here, we show that diverse amnestic treatments, such as concussion, commonly disrupt the presynaptic clustering of Synapsin, which is required for anesthesia-sensitive memory. Consistently, targeted knockout of *synaptojanin*, a key regulator of the endocytic pathway, selectively impaired labile memory and the Synapsin-associated vesicles. In contrast, we identified Rab3, a small GTPase that regulates the late steps of vesicle exocytosis at the active zone, as selectively required for anesthesia-resistant memory. Rab3 hyperactivation enhanced its association with vesicles while displacing Synapsin. Strikingly, this manipulation biased memory toward stabilization at the cost of the labile component. We thus propose distinct vesicle pools at the presynaptic terminal underlie the formation of labile and consolidated memories. Thus, our work offers a molecular framework for controlling memory stability through targeted manipulation of vesicle dynamics.

## Significance

What determines memory stability? We show that distinct presynaptic vesicle populations underlie the labile and consolidated memories, which are simultaneously formed following odor-shock associative learning in fruit flies. These distinct synaptic mechanisms explain the selective loss of labile memory triggered by traumatic perturbations after learning. Given the evolutionarily conserved regulators of vesicle dynamics, our findings provide insights into memory consolidation across species.

Memories can follow distinct fates—stabilization through consolidation or loss through forgetting. Although the concept of consolidated memory often refers to protein-synthesis-dependent long-term memory (LTM) induced by interspaced repeated training (1), it was originally established through resistance to retrograde amnesia (2). In *Drosophila melanogaster*, single-cycle aversive olfactory conditioning similarly induces two coexisting memory components: a labile form that decays within the first hours and is sensitive to brief post-training anesthesia induced by cold shock (anesthesia-sensitive memory [ASM]) and stable anesthesia-resistant memory (ARM) that persists after amnestic treatments (1, 3, 4). Unlike LTM, ARM can be formed after a single training trial and lasts more than 24 h, but does not require de novo protein synthesis (1, 4–7). How the memory trace for ARM is stabilized without changes in gene expression remains an intriguing question.

The neuronal circuits and molecular mechanisms underlying ASM and ARM have been intensely studied in the past decades (8, 9). Both memories involve the neurons in the mushroom body (MB) circuit (7, 10–14). Notably, memory-relevant plasticity is formed in the presynaptic terminals of Kenyon cells (KCs), the major intrinsic neurons of the MB (15–19). ARM and ASM involve distinct genes in KCs, such as different regulators of cAMP signaling, suggesting independent plasticity mechanisms (1, 7, 10, 20–25). Interestingly, many of these genes encode presynaptic proteins with distinct localization and functions in regulating neurotransmission. Notably, ARM involves the active-zone scaffold protein Bruchpilot (Brp), while Synapsin, a synaptic vesicle (SV) protein, and Tomosyn, a SNARE-binding protein, are selectively required for ASM (26–28). Collectively, distinct processes of the SV cycle are postulated to underlie plasticity for ARM and ASM in the KC presynaptic terminals.

To address this question, we focused on the presynaptic processes during the selective disruption of ASM in retrograde amnesia. Interestingly, chemical anesthetics, such as isoflurane, have been reported to inhibit SV exocytosis by altering the functions of specific synaptic proteins (29–31). However, it remains unclear if anesthesia by low temperature and chemical anesthetics induces the same form of amnesia—the loss of ASM. As a variety of perturbations, such as chemical anesthesia by ether, CO_2_, or N_2_, and experimental concussion (32–35), have been reported to induce retrograde amnesia, we investigate if ASM is generally susceptible to retrograde amnesia. Focusing on the anesthesia-sensitive presynaptic processes, we here show that the regulation of the distinct SV pools underlies labile and consolidated memories.

## Results

### Different Amnestic Treatments Disrupt the Same Memory Component.

**Fig. 1.**
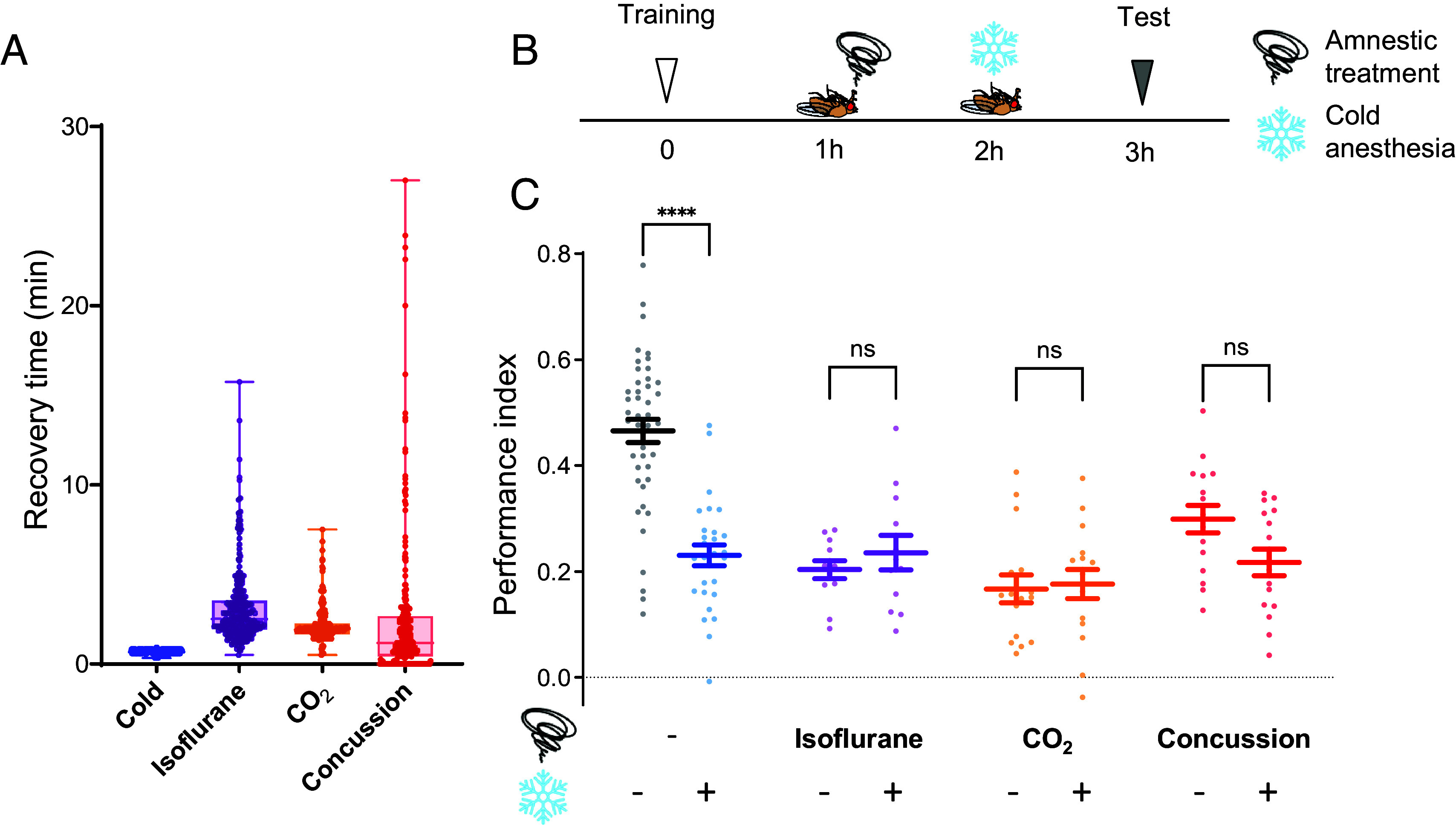
Various amnestic treatments affect the same labile memory component. (*A*) The recovery time until resumption of movement was measured for *Canton-S* flies following each type of anesthesia and concussion. Each data point represents recovery time of individual flies (N = 214-245). Box plots represent the median as the middle line, 1st and 3rd quartile as box boundaries. The whiskers extend to the minimum and maximum values of the dataset. (*B* and *C*) *Canton-S* flies were tested for 3-h aversive memory under various conditions: 2-min isoflurane anesthesia, 2-min CO_2_ anesthesia, concussion, or no treatment administered 1 h after training. Subsequently, flies were either subjected to additional 2-min cold anesthesia or left untreated for 2 h until test. Training consisted of pairing an odor (CS+) with an electric foot shock (US), followed by the presentation of a second odor (CS−) in the absence of a shock. Mean ± SEM are shown. One-way ANOVA, followed by multiple *t* tests, was performed (N = 12-44). Statistical summary for (*C*): One-way ANOVA showed a significant difference (*F* = 22.52, ****P < 0.0001). Multiple *t* tests with Bonferroni correction revealed significant differences between Control vs. Cold (*t* = 8.396, ****P < 0.0001), vs. Isoflurane (*t* = 6.944, ****P < 0.0001), vs. Isoflurane + Cold (*t* = 6.099, ****P < 0.0001), vs. CO_2_ (*t* = 8.828, ****P < 0.0001), vs. CO_2_ + Cold (*t* = 8.559, ****P < 0.0001), vs. Concussion (*t* = 4.922, ****P < 0.0001), and vs. Concussion + Cold (*t* = 7.349, ****P < 0.0001). The following comparisons were not significant: Isoflurane vs. Isoflurane + Cold (*t* = 0.674, *P* > 0.9999; n.s.), CO_2_ vs. CO_2_ + Cold (*t* = 0.2215, *P* > 0.9999; n.s.), Concussion vs. Concussion + Cold (*t* = 2.004, *P* = 0.7501; n.s.), Isoflurane vs. Cold (*t* = 0.6716, *P* > 0.9999; n.s.), CO_2_ vs. Cold (*t* = 1.747, *P* > 0.9999; n.s.), Concussion vs. Cold (*t* = 1.891, *P* = 0.9676; n.s.), Cold vs. Isoflurane + Cold (*t* = 0.126, *P* > 0.9999; n.s.), Cold vs. CO_2_ + Cold (*t* = 1.497, *P* > 0.9999; n.s.), and Cold vs. Concussion + Cold (*t* = 0.3691, *P* > 0.9999; n.s.).

ASM and ARM coexist several hours after single-cycle aversive olfactory conditioning in *Drosophila* (1). To address if ASM disrupted by cold anesthesia represents general retrograde amnesia, we examined 3-h memory with different types of post-training perturbations: gaseous anesthesia with CO_2_ and isoflurane, and experimental concussion. These perturbations have been previously characterized as triggers for retrograde amnesia in other animals (32–35). We confirmed that these treatments induce paralysis as quickly as cold anesthesia and allow flies to recover within 5 min (Fig. 1*A*). We found that anesthesia by CO_2_ and isoflurane, and experimental concussion applied 1 h after training reduced 3-h aversive memory to a similar extent as cold anesthesia (Fig. 1 *B* and *C*). If these perturbations disrupt a different memory component from ASM by cold anesthesia, the amnestic effect should be additive when cold anesthesia is applied subsequently. Additional cold anesthesia, however, did not further reduce the memory score after isoflurane, CO_2_ anesthesia, or concussion (Fig. 1 *B* and *C*). Thus, ASM is generally sensitive to retrograde amnesia irrespective of perturbations—likely through the same anesthesia-sensitive mechanism.

### Amnestic Treatments Reduce the Synapsin Clusters in the Presynaptic Boutons.

Next, we sought to identify the molecular target of retrograde amnesia that is commonly affected by various amnestic treatments. As a plausible target, we focused on the localization of the presynaptic proteins. Given the selective requirement of Synapsin and Brp for ASM and ARM, respectively (27, 36), amnestic perturbations may have differential impacts on these two proteins.

To examine the subcellular localization of Synapsin and Brp, we took advantage of the large presynaptic terminals of the olfactory projection neurons (PNs) in the MB calyx. As the calyx microcircuits consist of terminals from different neurons (37–39), we selectively labeled endogenous Synapsin and Brp proteins in PNs using a CRISPR/Cas9-mediated split-GFP tagging strategy (40, 41). We inserted the GFP_11_ tag at the C-terminal of Synapsin (see Methods for details) and Brp (41), and directed GFP reconstitution in PNs with *GH146-GAL4* and *UAS-GFP_1-10_*. We confirmed barely detectable background signals with either GFP_11_ tags or GFP_1-10_ alone (*SI Appendix*, Fig. S1). Reconstituted GFP (rGFP) signals of Synapsin were highly localized to PN boutons, appearing as focal clusters.

**Fig. 2.**
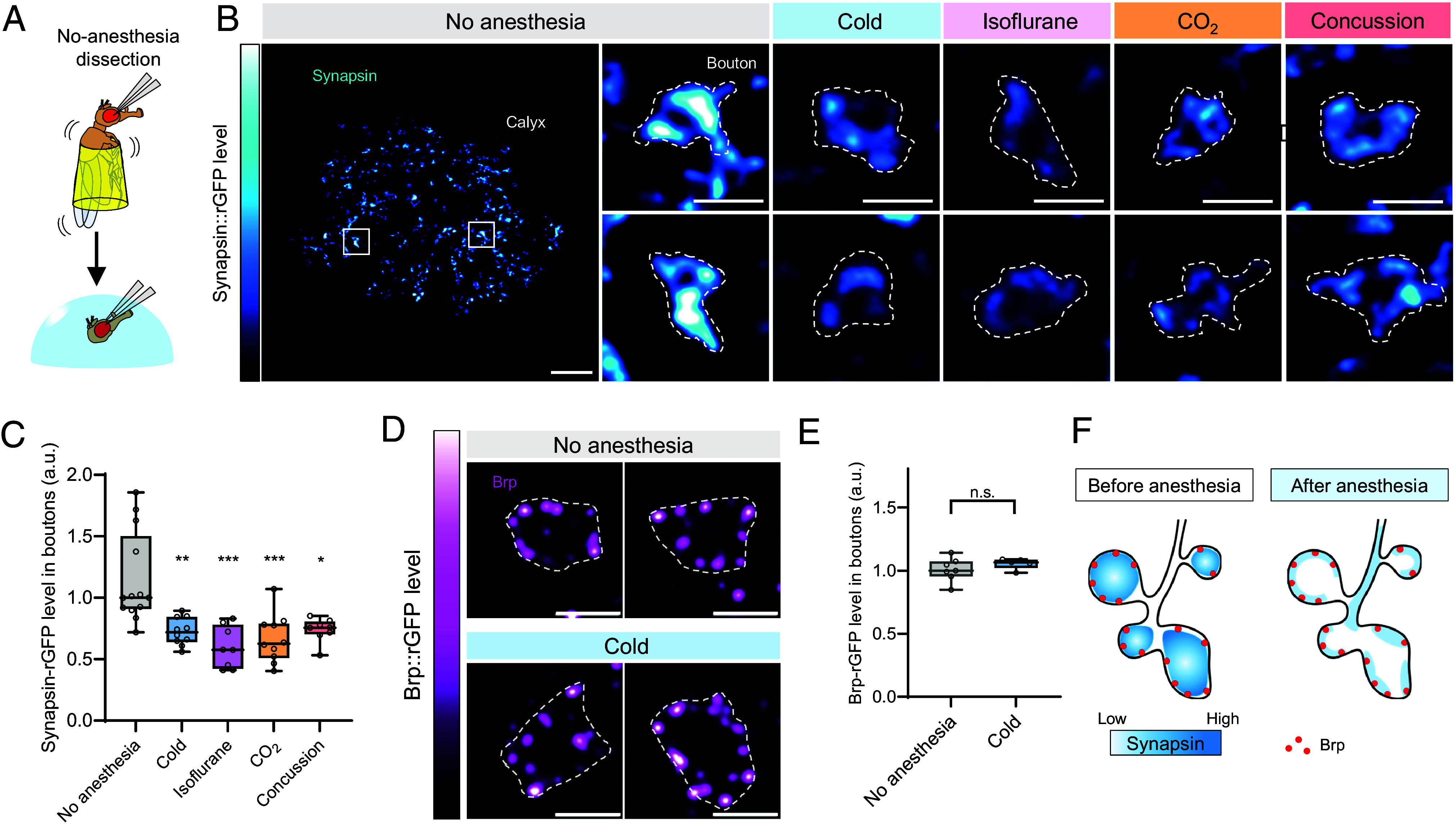
Amnestic treatments selectively reduce Synapsin in the presynaptic boutons of the olfactory projection neurons. (*A*) As an experimental control, fly brains were dissected without anesthesia (“No-anesthesia” group). (*B* and *C*) Single plane confocal images showing Synapsin::rGFP in PN boutons in the MB calyx from flies after 2-min cold-, isoflurane-, or CO_2_-anesthesia, concussion, or no treatment. *GH146-Gal4* was used to express *UAS-GFP_1-10_* in PNs. Quantification of Synapsin::rGFP levels in PN boutons revealed a significant decrease following anesthesia or concussion. The mean of 20 boutons from each calyx were measured to represent each brain sample. Kruskal–Wallis test followed by Dunn’s multiple comparison was performed (N = 8-12). (*D* and *E*) Single plane confocal images showing Brp::rGFP in PN boutons in MB calyx from flies with or without 2-min cold anesthesia. *GH146-Gal4* was used to express *UAS-GFP_1-10_* in PNs. Quantification of Brp::rGFP levels in PN boutons showed no significant changes following cold anesthesia. The mean of 10 boutons from each calyx were measured to represent each brain sample. Unpaired *t* test was performed (N = 5-7). (*F*) Schematics of Synapsin and Brp localization in PN boutons before and after anesthesia. Scale bar, 10 µm (*B*, *Top*), 2 µm (*B*, *Bottom*, and *D*). Pixel intensities were normalized with the median values of the control group. Box plots represent the median as the middle line, 1st and 3rd quartile as box boundaries. The whiskers extend to the minimum and maximum values of the dataset. Statistic summary for (*C*): Kruskal–Wallis test showed a significant difference (*H* = 23.89, ****P < 0.0001). Dunn’s multiple comparisons test revealed significant differences between No anesthesia vs. Cold (**P = 0.0068), vs. Isoflurane (****P = 0.0001), vs. CO_2_ (***P = 0.0005), and vs. Concussion (*P = 0.0286). (*E*): Mann–Whitney *U* test showed no significant difference between No anesthesia vs. Cold (*P* = 0.3434; n.s.).

To evaluate the effect of anesthesia, we prepared brain samples from flies without anesthesia and compared them with those treated using the respective amnestic methods (Fig. 2*A*). Strikingly, cold anesthesia significantly reduced Synapsin::rGFP, with only faint signals remaining around the periphery of the boutons (Fig. 2 *B* and *C*). In contrast, the levels of Brp::rGFP in the PN terminals were unaffected by cold anesthesia (Fig. 2 *D* and *E*), identifying Synapsin as the selective presynaptic target of anesthesia (Fig. 2*F*). To further validate the Synapsin::rGFP reduction after cold anesthesia, we confirmed this effect using anti-Synapsin staining and alternative dissection method (*SI Appendix*, Fig. S2). Moreover, the reduction of Synapsin::rGFP signals was consistent across different amnestic treatments (Fig. 2 *B* and *C*). These results suggest that anesthesia-induced dispersion of Synapsin clusters serves as a molecular hallmark of retrograde amnesia. Considering the molecular functions of Synapsin in clustering SVs to form the reserve pool (RP) (42–44), anesthesia may disrupt specific steps of the SV cycle.

### Disruption of the Selective Endocytic Pathway Impairs the Synapsin-Associated SV Pool and Labile Memory.

**Fig. 3.**
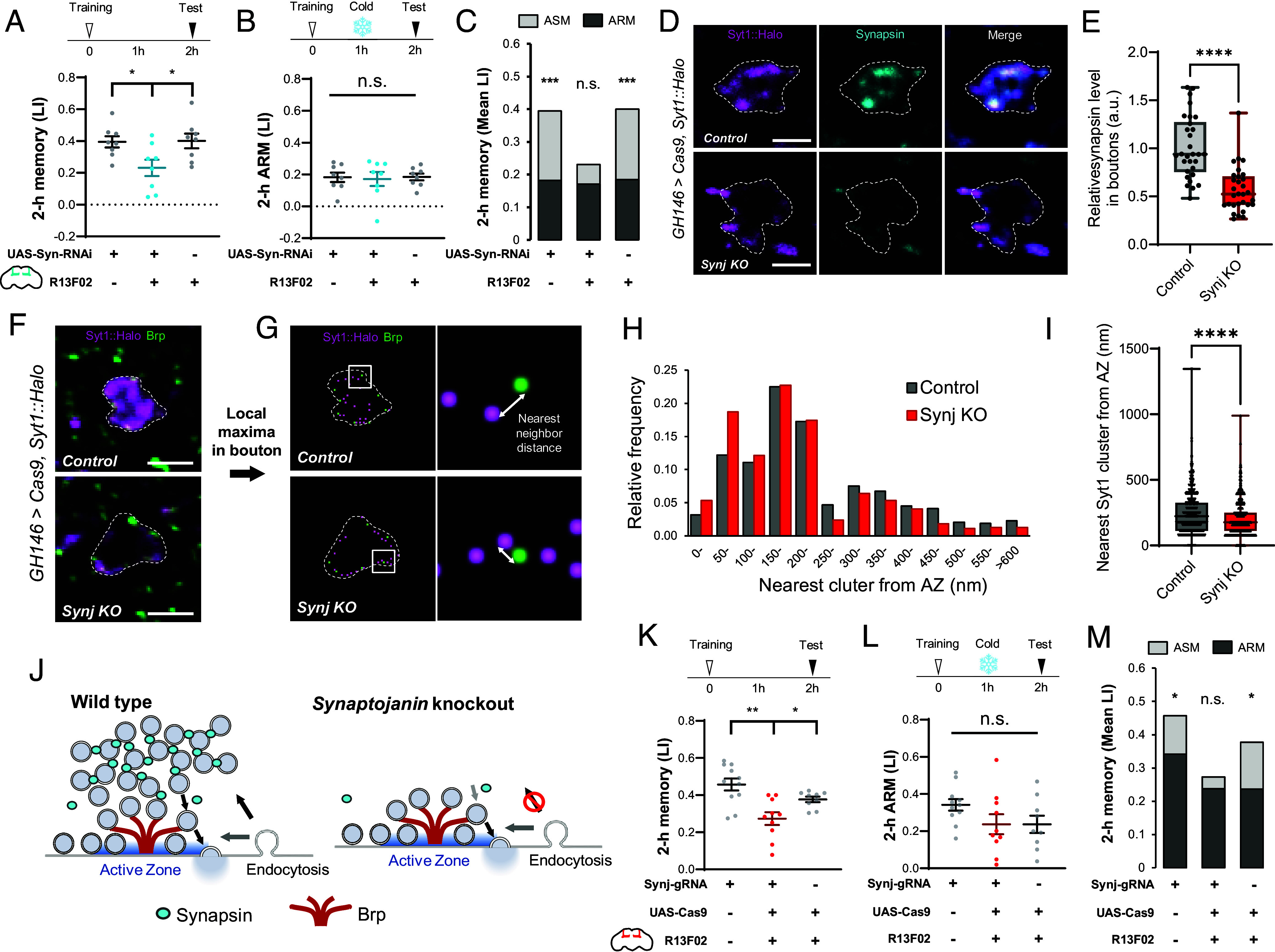
*Synaptojanin* knockout impairs Synapsin SV formation and the labile memory. (*A*–*C*) *Synapsin* was knocked down in KCs using *R13F02-GAL4*, a KC-specific driver. Flies were tested for 2-h aversive olfactory memory with (*B*) or without (*A*) 2-min cold anesthesia 1 h after training. (*C*) ASM score, difference between the mean total memory and ARM scores, is reduced upon *synapsin* knockdown, resulting in no significant difference between total memory and ARM. N = 8. (*D*–*I*) *synj* was knocked out in the PNs by expressing *UAS-u^M^-Cas9* using *GH146-GAL4* together with the guide RNA for *synj* mutagenesis under control of the ubiquitous U6 promoter. SVs were visualized by expressing *UAS-Syt1::7xHalo7* as a marker (magenta). Brains were immuno-stained using anti-Synapsin (*D*, cyan) or Brp (*F*, green) antibodies. Panels *D* and *F* show representative presynaptic boutons in the MB calyx (circled with dashed lines). (Scale bar, 2 µm.) (*E*) Quantification of Synapsin levels in PN boutons revealed significant decrease in *synj* KO boutons. 10 boutons were measured from three calyces from different brain samples. Mann–Whitney *U* test was performed (N = 30; ****P < 0.0001). (*G*–*I*) The distance to Syt1 cluster from each Brp peak is measured by calculating the nearest-neighbor distance between local maxima points of Syt1::Halo and Brp signals. (*G*) exemplifies local maxima of green (anti-Brp) and magenta (Syt1::Halo) signals detected from the same boutons shown in (*F*). Distance of the nearest Syt1 clusters from each Brp peak in control and *synj* KO boutons (533 and 545 AZs are analyzed, respectively). (*H*) Relative frequency histogram. (*I*) Median distance is significantly reduced in *synj* KO boutons (Mann–Whitney *U* test; ****P < 0.0001). (*J*) Schematics of the selective disruption of Synapsin-positive SV cluster formation in Synaptojanin KO synapses. (*K*–*M*) *synj* was knocked out in KCs using *UAS-u^S^-Cas9* with *R13F02-GAL4*, a KC-specific driver. Flies were tested for 2-h aversive olfactory memory with (*L*) or without (*K*) 2 min cold anesthesia 1 h after training. (*M*) ASM score, difference between the mean total memory and ARM scores, is reduced in the *synj* KO group, revealing no significant difference between total memory and ARM. N = 9-11. Mean ± SEM are shown in (*A*, *B*, *K*, and *L*). Box plots in (*E* and *I*) represent the median as the middle line, 1st and 3rd quartile as box boundaries. The whiskers extend to the minimum and maximum values of the dataset. Statistical summary for (*A*): One-way ANOVA showed a significant difference (*F* = 4.579, *P = 0.0224). Multiple *t* test with Benjamini and Hochberg correction revealed significant differences between CS × UAS-Syn-RNAi vs. *R13F02* × *UAS-Syn-RNAi* (*P = 0.0177), and R13F02 × UAS-Syn-RNAi vs. *R13F02 × CS* (*P = 0.0144). (*B*): One-way ANOVA showed no significant difference (*F* = 0.04415, *P* = 0.9569; n.s.). Multiple *t* test with Benjamini and Hochberg correction showed no significant differences between *CS × UAS-Syn-RNAi* vs. *R13F02 × UAS-Syn-RNAi* (*P* = 0.8213; n.s.) and *R13F02 × UAS-Syn-RNAi* vs. *R13F02 × CS* (*P* = 0.7833; n.s.). (*C*) Multiple *t* test with Benjamini and Hochberg correction revealed significant difference between the mean total memory and ARM in *CS × UAS-Syn-RNAi* (***P = 0.0004) and *R13F02 × CS* (***P = 0.0008), but not in *R13F02 × UAS-Syn-RNAi* (*P* = 0.3975; n.s.). (*K*): One-way ANOVA showed a significant difference (*F* = 10.09, ***P = 0.0005). Multiple *t* test with Benjamini and Hochberg correction revealed significant differences between *Synj-gRNA × CS* vs. *Synj-gRNA × R13F02>Cas9* (***P = 0.0001), and *Synj-gRNA × R13F02>Cas9* vs. *CS × R13F02>Cas9* (*P = 0.0234). (*L*): One-way ANOVA showed no significant difference (*F* = 1.968, *P* = 0.1238; n.s.). Multiple *t* test with Benjamini and Hochberg correction showed no significant differences between *Synj-gRNA × CS* vs*. Synj-gRNA × R13F02>Cas9* (*P* = 0.0974; n.s.) and *Synj-gRNA × R13F02>Cas9* vs. *CS × R13F02>Cas9* (*P* = 0.9947; n.s.). (*M*) Multiple *t* test with Benjamini and Hochberg correction revealed significant differences between the mean total memory and ARM in *Synj-gRNA × CS* (*P = 0.0269) and *CS × R13F02>Cas9* (*P = 0.0343), but not *in Synj-gRNA × R13F02>Cas9* (*P* = 0.5755; n.s.).

First, we tested the role of Synapsin in KCs and confirmed that KC-specific knockdown reduced ASM while leaving ARM intact (Fig. 3 *A*–*C* and *SI Appendix*, Fig. S3 *A* and *B*), a selective memory deficit as previously reported using a *synapsin* mutant (36). The requirement of Synapsin for ASM, along with the anesthesia-induced reduction, suggests the regulation of a specific SV pool underlying labile memory. To address this, we focused on Synaptojanin (Synj), a widely conserved phosphoinositide phosphatase (45, 46). Loss of *synj* was reported to reduce SV clustering in the presynaptic terminals by inhibiting the specific endocytic pathways that constitute the recycling vesicle pool (47–49).

Using cell-type specific CRISPR-mediated mutagenesis (50–52), we knocked out *synj* in PNs using *GH146-GAL4* and characterized the localization of Synapsin and the SV marker Syt1::Halo (53). As in the post-anesthesia state (Fig. 2 *B* and *C*), *synj* mutation greatly reduced SVs and Synapsin in the boutons (Fig. 3 *D* and *E*). SVs and Synapsin in the *synj^−^* boutons remained at the periphery, near the AZ (Fig. 3*F*). Localization analysis revealed that Syt1 signals were shifted closer to Brp in *synj^−^* boutons, suggesting altered SV cluster organization (Fig. 3 *G*–*I*). These results show that the SV pool tethered at the AZ is largely unaffected by *synj* knockout, in line with the role of Synj in determining the endocytic pathways (49). Taken together, the *synj* knockout selectively deprives the Synapsin-associated SV clusters that are sensitive to anesthesia (Fig. 3*J*).

To examine the roles of the Synj-regulated SV pool in ASM, we measured 2-h aversive olfactory memory in flies with *synj* knockout specifically in KCs using *R13F02-GAL4*. The total memory of the KC-specific *synj* knockout flies was significantly impaired, whereas ARM after cold anesthesia remained intact (Fig. 3 *K* and *L*), selectively reducing ASM (Fig. 3*M*). We confirmed that innate avoidance of both the odor and the electric shock used in conditioning remained unaffected (*SI Appendix*, Fig. S3 *C* and *D*). The altered presynaptic SV distribution and the selective memory loss in the *synj* knockout flies together indicate that the Synapsin-associated SV pool undergoes the synaptic plasticity critical for labile memory.

### Rab3 Regulates Synaptic Vesicles at the AZ for ARM.

The selective requirement of Brp for ARM (27), along with the largely unaffected vesicles at the AZ in the *synj^-^* boutons (Fig. 3 *F*–*J*), suggests that plasticity of the SV pool at the AZ mediates ARM. As a plausible vesicle effector for this regulation, we focused on Rab3, a small GTPase that controls vesicle docking, fusion, and organizes the protein composition of AZs (54–57).

**Fig. 4.**
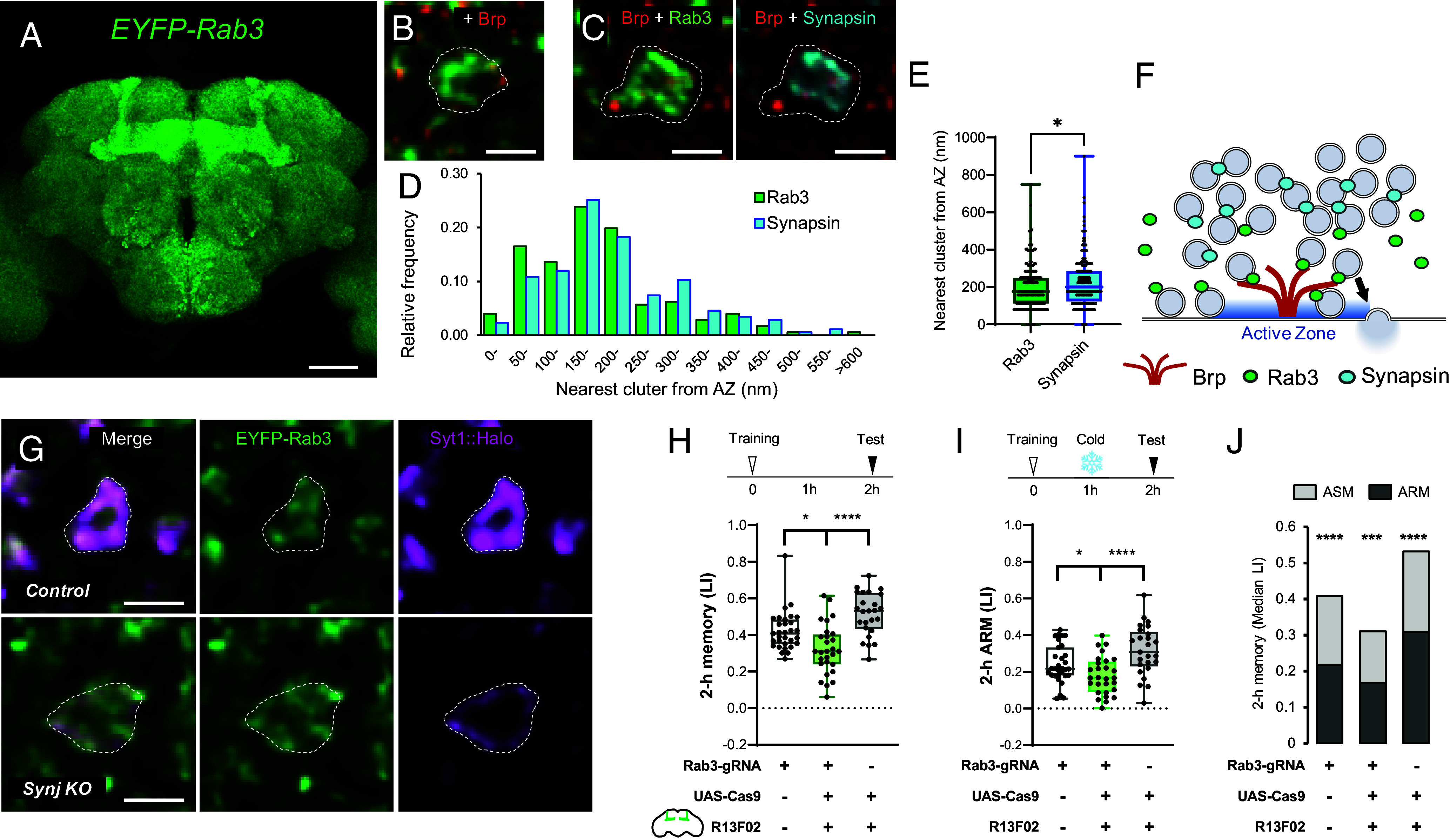
Rab3 exists on the vesicles at the AZ and contributes to the consolidated memory. (*A*–*E*) Localization of EYFP-Rab3 (green). (*A*) Max projection of the whole brain of an adult fly showing enrichment of Rab3 in the MB lobe. Colocalization of EYFP-Rab3 with anti-Brp immunostaining (*B*, red). (*C*–*E*) Localization of endogenously labeled Rab3 (green) and Synapsin (cyan) in the PN boutons. For cell-type-specific visualization, *B2RT-STOP-B2RT-mCherry-Rab3* and *Synapsin::GFP_11_* are used, and B2 recombinase and GFP_1-10_ are expressed by using *GH146-GAL4*. Nearest neighbor distances of local maxima of Rab3 and Synapsin signals from each AZ, local maxima of anti-Brp signals, are measured (176 AZs are analyzed). (*D*) Relative frequency histogram of nearest neighbor Synapsin and Rab3 maxima from Brp peaks. (*E*) Median distance of Rab3 is shorter than that of Synapsin (Mann–Whitney *U* test; *P = 0.0183). (*F*) Schematics of Rab3 localization on the vesicles at the AZ. (*G*) EYFP-Rab3 signals colocalize with the remaining vesicles (magenta) in the synj knockout synapses. *GH146-GAL4* was used to express *UAS-Syt1::7xHalo7* and *UAS-uM-Cas9* together with ubiquitously expressed *U6.2-synj-gRNA*. Single focal slices of representative presynaptic boutons of PNs in the MB calyx are shown in (*B*, *C*, and *G*). [Scale bar, 50 µm (*A*), 2 µm (*B*, *C*, and *G*).] (*H*–*J*) For Rab3 cell-type-specific knockout, *UAS-u^S^-Cas9* was expressed under control of KC-specific *R13F02-GAL4* or pan-neuronal *R57C10-GAL4* together with ubiquitously expressed *U6:3-Rab3-gRNA*. 2-h aversive olfactory memory was tested with (*I*) or without (*H*) 2 min cold anesthesia 1 h after training. (*J*) Cold anesthesia significantly reduced the memory score in all genotypes by a similar amount, suggesting intact ASM in these flies. N = 25-31. Box plots represent the median as the middle line, 1st and 3rd quartile as box boundaries. The whiskers extend to the minimum and maximum values of the dataset. Statistical summary for (*H*): Kruskal–Wallis test showed a significant difference (*H* = 24.81, ****P < 0.0001). Multiple Mann–Whitney *U* test with Benjamini and Hochberg correction revealed significant differences between *Rab3-gRNA × yw* vs. *Rab3-gRNA × R13F02>Cas9* (*P = 0.0108), and *Rab3-gRNA × R13F02>Cas9* vs. *w × R13F02>Cas9* (****P < 0.0001). (*I*): Kruskal–Wallis test showed a significant difference (*H* = 15.15, ***P = 0.0005). Multiple Mann–Whitney *U* tests with Benjamini and Hochberg correction revealed significant differences between *Rab3-gRNA × yw* vs. *Rab3-gRNA × R13F02>Cas9* (*P = 0.0457), *and Rab3-gRNA × R13F02>Cas9* vs. *w × R13F02>Cas9* (****P < 0.0001). (*J*) Multiple Mann–Whitney *U* test with Benjamini and Hochberg correction revealed significant difference between the mean total memory and ARM in all three genotypes: *Rab3-gRNA × yw* (****P < 0.0001), *Rab3-gRNA × R13F02>Cas9* (***P = 0.0001), and *w × R13F02>Cas9* (****P < 0.0001).

Consistent with Rab3’s presynaptic functions, endogenously labeled EYFP::Rab3 (58) was expressed in neuropils with a notable elevation in the MB lobes (Fig. 4*A*). Interestingly, EYFP::Rab3 was enriched around the AZ (Fig. 4*B*). Further analysis of Rab3 and Synapsin localization within the same boutons revealed that Rab3 is positioned closer to the AZ, suggesting spatial segregation (Fig. 3 *C*–*F*). In contrast to Synapsin (Fig. 3 *D* and *E*), Rab3 remained localized to the vesicles in the *synj^-^* boutons (Fig. 4*G*). These anatomical results imply that Rab3 mediates ARM-related plasticity via AZ-tethered vesicles, distinct from the Synapsin-associated SV pool for ASM.

To test this hypothesis, we measured 2-h aversive olfactory memory in flies with KC-specific CRISPR-mediated knockout of *rab3* by expressing Cas9 using *R13F02-GAL4*, respectively. In *rab3* knockout flies, ARM measured after post-training cold anesthesia was significantly reduced (Fig. 4 *H* and *I*), whereas ASM remained unaffected (Fig. 4*J*). The KC-specific knockout did not show any significant effects on the avoidance of either electric shock or odor (*SI Appendix*, Fig. S3 *E* and *F*), indicating normal sensory and locomotor functions. Given that Rab3 is specifically required for ARM, we conclude that Rab3 in KCs mediates presynaptic plasticity for consolidated memory.

### Rab3 Hyperactivation Shifts the Molecular Composition in SVs and Increases the Anesthesia Resistance of Memory.

**Fig. 5.**
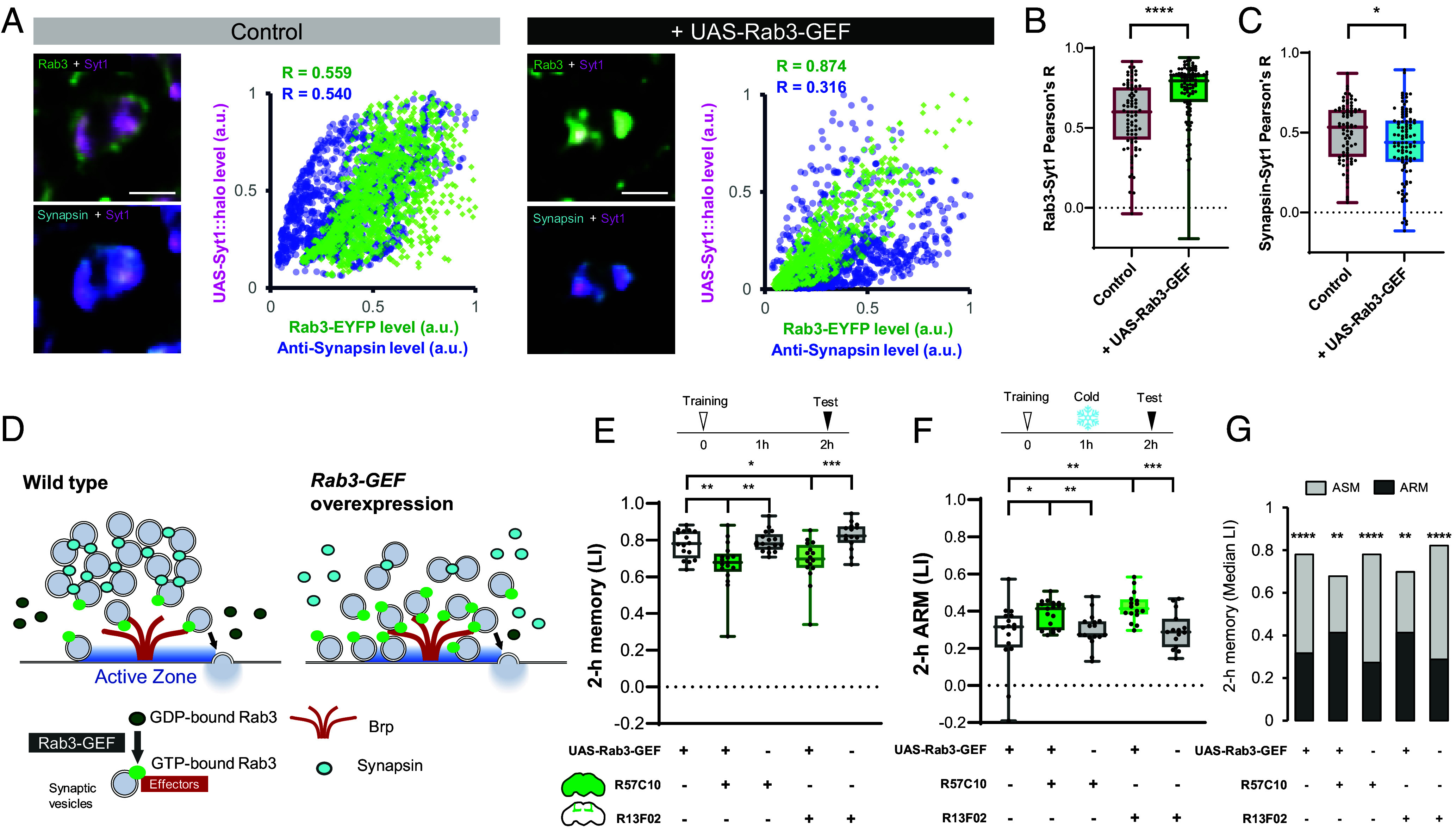
Rab3 hyperactivation using *UAS-Rab3-GEF* changes the composition of SV molecules and increases the anesthesia resistance of memory. (*A*) Colocalization analysis of EYFP-Rab3 (green) and anti-Synapsin (cyan) with Syt1::7xHalo7 (magenta), an SV marker. *GH146-GAL4* was used to express *UAS-Syt1::7xHalo7* and *UAS-Rab3-GEF*. Representative presynaptic boutons of PNs in the MB calyx are shown. Scatter plots were generated from the shown boutons, with pixel intensities normalized by the maximum values in each channel. The Pearson correlation coefficient (R), calculated from the colocalization plots, is displayed on the upper left with corresponding colors. (Scale bar, 2 µm.) (*B* and *C*) To quantify the colocalization of Rab3 and Synapsin with SVs, Pearson’s R based on pixel intensities was calculated as in panel *B* From each brain, 20 boutons were analyzed from calyces of 4-5 different brain samples. Mann–Whitney *U* test was performed (N = 80-100). Box plots represent the median as the middle line, 1st and 3rd quartile as box boundaries. The whiskers extend to the minimum and maximum values of the dataset. Statistic summary for (*B*): Mann–Whitney *U* test revealed a significant difference between Control vs. +UAS-Rab3-GEF (****P < 0.0001). (*C*): Mann–Whitney *U* test showed a significant difference between Control vs. +UAS-Rab3-GEF (*P = 0.0396). (*D*) Schematics summarizing the results indicating increased Rab3 vesicles and decreased Synapsin-associated SV pool upon overexpression of Rab3-GEF. (*E*–*G*) UAS-Rab3-GEF was expressed using KC-specific *R13F02-GAL4* or pan-neuronal *R57C10-GAL4*. 2-h aversive olfactory memory was tested without (*E*) or with (*F*) 2 min cold anesthesia 1 h after training. (*G*) Cold anesthesia significantly reduced the memory score in all genotypes, but by smaller degree in the flies with Rab3-GEF overexpression. N = 15-17. Box plots represent the median as the middle line, 1st and 3rd quartile as box boundaries. The whiskers extend to the minimum and maximum values of the dataset. n.s.: *P* > 0.05, *P < 0.05, **P < 0.01, ***P < 0.001, ****P < 0.0001. Statistical summary for (*E*): Kruskal–Wallis test showed a significant difference (*H* = 26.43, ****P < 0.0001). Multiple Mann–Whitney *U* test with Benjamini and Hochberg correction revealed significant differences between *Rab3-GEF × CS* vs. *Rab3-GEF × R57C10* (**P = 0.0076), *Rab3-GEF × R57C10* vs. *CS × R57C10* (**P = 0.0015), *Rab3-GEF × CS* vs. *Rab3-GEF × R13F02* (*P = 0.0237), and *Rab3-GEF × R57C10* vs. *CS × R13F02* (***P = 0.0001). (*F*): Kruskal–Wallis test showed a significant difference (*H* = 21.58, ***P = 0.0002). Multiple Mann–Whitney *U* test with Benjamini and Hochberg correction revealed significant differences between *Rab3-GEF × CS* vs. *Rab3-GEF × R57C10* (*P = 0.0279), *Rab3-GEF × R57C10* vs. *CS × R57C10* (**P = 0.0091), *Rab3-GEF × CS* vs. *Rab3-GEF × R13F02* (**P = 0.0015), and *Rab3-GEF × R57C10* vs. *CS × R13F02* (***P = 0.0006). (*G*) Multiple Mann–Whitney *U* test with Benjamini and Hochberg correction revealed significant difference between the mean total memory and ARM in all five genotypes: *Rab3-GEF × CS* (****P < 0.0001), *Rab3-GEF × R57C10* (**P = 0.0025), *CS × R57C10* (****P < 0.0001), *Rab3-GEF × R13F02* (**P = 0.008), and *CS × R13F02* (****P < 0.0001).

The Rab3-SV association may be crucial for regulating the magnitude of ARM. The interaction between Rab3 and SVs is known to be activated upon GTP binding (59–61). To manipulate this interaction, we hyperactivated Rab3 by overexpressing *Rab3-GEF* (61). First, we characterized SV molecular composition and their spatial distribution in the presynaptic boutons of PNs by simultaneously visualizing Rab3, Synapsin, and Syt1, an SV marker. Intriguingly, *Rab3-GEF* expression visibly altered the localization of both Rab3 and Synapsin within boutons (Fig. 5*A*). To quantify colocalization of Rab3 and Synapsin with SVs marked with Syt1::Halo, we calculated Pearson’s correlation coefficient of signal intensities within each bouton (Fig. 5 *A*–*C*). As a result, *Rab3-GEF* expression enhanced the colocalization of Rab3 with the SV marker (Fig. 5*B*). In contrast, Synapsin-SV colocalization was significantly decreased (Fig. 5*C*). Collectively, these results reveal a shift in the molecular composition on SVs upon Rab3 hyperactivation (Fig. 5*D*).

Next, we examined the memory composition upon Rab3 activation. Strikingly, overexpression of *Rab3-GEF* with either pan-neuronal or KCs-specific GAL4 improved 2-h ARM while reducing total memory (Fig. 5 *E*–*G*). As a result, calculated ASM was greatly reduced with *Rab3-GEF* overexpression (Fig. 5*G*). *Rab3-GEF* expression in KCs was sufficient to induce the effect comparable to pan-neuronal expression (Fig. 5 *E* and *F*), which aligns with the major contribution of KCs to both ARM and ASM. We confirmed that sensitivity to electric shock or odor was unaffected (*SI Appendix*, Fig. S3 *G* and *H*). Based on the functions of Rab3 (Fig. 4) and Synapsin (36) in memory, the altered SV molecular composition may have strengthened ARM at the cost of ASM.

## Discussion

### Loss of Labile Memory through Synapsin SV Clusters.

By using volatile anesthetic chemicals and experimental concussion in addition to cold anesthesia, we demonstrated a common perturbation of ASM (Fig. 1 *B* and *C*). Traditionally, studies on retrograde amnesia have relied on a single amnestic treatment, making it difficult to discover universal mechanisms. In *Drosophila*, cold anesthesia has been routinely applied since the first report in 1976 due to rapid recovery (Fig. 1*A*) and minimal harm to insects (3). In parallel with the common effect on memory, all tested treatments consistently reduced Synapsin from PN terminals (Fig. 2). Although Synapsin localization is conserved across different cell types and systems (43, 44, 62, 63), superresolution approaches may clarify the localization within the narrow KC terminals. While many molecular targets of anesthetics have been identified (29, 30, 64, 65), a common target has not yet been implicated. Considering the behavioral function of Synapsin (36), our results suggest that Synapsin is a molecular target underlying retrograde amnesia. How such diverse amnestic treatments—ranging from cold exposure to chemical anesthetics and mechanical shocks—all converge on inducing Synapsin dispersion remains unknown. Since Ca^2+^ signaling and endo/exocytosis regulation were shown to influence Synapsin localization (66, 67), these synaptic processes may be commonly disrupted during retrograde amnesia.

**Fig. 6.**
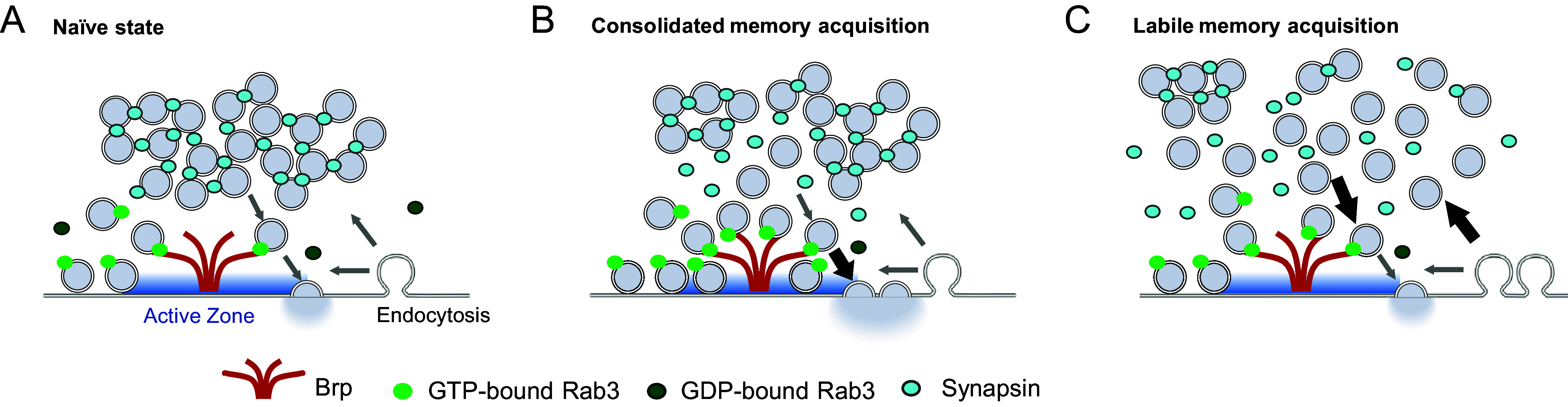
A working model of plasticity for consolidated and labile memory involving Rab3 and Synapsin. (*A*) In the naive state, the Synapsin-positive vesicle clusters and the Rab3-positive vesicles at the AZ are maintained by SV cycling. (*B*) Consolidated memory may involve facilitation of exocytosis at the AZ, driven by GTP-bound Rab3, which promotes vesicle recruitment, priming, and AZ remodeling. (*C*) The acquisition of labile memory may depend on RP vesicle dynamics, governed by Synapsin-mediated regulation, potentially through endocytosis, actin polymerization, and Synapsin phosphorylation.

The mechanism of labile memory loss offers insight into its formation. Prior studies show that *synapsin* and *synj* mutants exhibit the selective reduction of the reserve pool (RP), an SV population for sustained neurotransmission during prolonged high-frequency stimulation (43, 46, 47, 68). We propose three possible scenarios for how ASM-related plasticity is induced in the RP. First, a specific endocytic pathway required for RP formation (69, 70) may undergo plastic changes during the acquisition of ASM (Fig. 6 *A* and *C*). This scenario is plausible considering the function of *synj* in regulating the RP size via endocytosis (49, 70). Consistently, Synapsin loading to SVs takes place at the endocytic zone after prolonged stimulation (63, 71). Second, actin dynamics in presynaptic terminals may also contribute to plasticity through the formation and vesicle mobilization in the RP (63, 72, 73). Supporting this, actin polymerization by Rac1 accelerates gradual forgetting of labile memory in *Drosophila* (74, 75). Third, regulation of RP vesicle mobility can account for the plasticity possibly through changing the phosphorylation states of Synapsin (66, 76–81) (Fig. 6 *A* and *C*). Memory traces in the RP are likely reset upon anesthesia-induced Synapsin dispersion (Fig. 2), leading to retrograde amnesia, as these regulatory mechanisms depend on Synapsin (42, 44, 62, 63, 71, 80, 81). Given that the amnestic treatments used here also induce retrograde amnesia in other species (32–35), these molecular mechanisms may be conserved across species.

### Plasticity in the Vesicle Pool at the AZ Underlies Consolidated Memory.

Our findings from Rab3 knockout (Fig. 4 *H*–*J*) and Rab3-GEF overexpression (Fig. 5 *E*–*G*) support Rab3 as a key player in presynaptic plasticity for ARM. The localization of Rab3 (Fig. 4 *B*–*F*) suggests its function at the AZ cytomatrix. Consistently, Brp is selectively required for ARM (27), and the Brp clusters at the AZ remained unaltered after anesthesia (Fig. 2 *D* and *E*). In addition, the role of Rab3 in the assembly of the AZ cytomatrix explains learning-induced Brp remodeling in KCs (56, 82–84). Therefore, we propose that the Rab3-regulated plasticity in the SV pool at the AZ underlies consolidated memory (Fig. 6*B*).

SVs at the AZ constitute the readily releasable pool (RRP) (85). Rab3 regulates RRP by controlling its size and release probability, two key physiological parameters for plasticity (54, 55, 57, 86). Rab3’s functions in the recruitment of SVs to the AZ (54, 60), vesicle priming (55, 87), and AZ remodeling (56, 57) provide multiple possible mechanisms for the plasticity involving the RRP vesicles (Fig. 6 *A* and *B*). Together with the role of RP regulation for labile memory (Fig. 6), we propose distinct forms of plasticity in presynaptic vesicle pools underlying labile and consolidated memory components. As well as these functions in mature neurons, loss of any presynaptic machinery could affect development, and adult-specific and chronic knockdown or knockout may reveal differential morphological and behavioral phenotypes, as exemplified by *Bruchpilot* knockdown (27, 88). Therefore, measuring both RP and RRP dynamics in these flies with selective memory deficits would be promising to reveal plasticity mechanisms in the corresponding pools.

### Differential Molecular Compositions of SV Pools Underlying the Labile and Consolidated Memories.

Our results suggest that Synapsin and Rab3 are two key regulators of vesicle dynamics for labile and consolidated memories, respectively (Fig. 6). These differentially regulated vesicles correspond to physiologically defined pools with distinct release kinetics: RRP vesicles are released immediately upon high-frequency stimulation, while RP vesicles are mobilized during prolonged stimulation (89). The release modes of these pools can explain the priority of the consolidated memory component ARM, which can be instantly acquired with a single electric shock pulse (36, 90). In contrast, the formation of ASM requires intense or repeated electric shocks (36, 90). Therefore, a single presynaptic terminal of a KC can in principle accommodate two distinct forms of plasticity in different vesicle pools for ASM and ARM (Fig. 6). As previous studies reported differential contributions of KC subtypes to ASM or ARM (10, 11, 13, 91), molecular and vesicular compositions of presynaptic terminals can vary among these neurons. Additionally, other neurons in the olfactory circuit, especially those modulating KC output, were shown to selectively contribute to ASM or ARM (7, 11, 13, 14, 91, 92). Since these neurons use different neuromodulators including dopamine, octopamine, and serotonin, they can preferentially contribute to plasticity in specific vesicle pools.

Interestingly, Synapsin and RP-like SV clusters were conserved in bilaterally symmetric animals, but only occasionally found in animals with simpler or no nervous systems, whereas Rab family proteins have more ancient evolutionary origins (93–95). This suggests that the large SV storage of Synapsin-regulated RP may have emerged later during evolution. The more complex presynaptic machinery could be adaptive by allowing multimodal plasticity with distinct vesicle pools.

Overexpression of *Rab3-GEF* demonstrates that the state of Rab3 determines the molecular composition of SVs (Fig. 5 *A*–*D*). This flexibility is supported by previously identified biochemical interaction between Synapsin and Rab3 on SVs (96). Manipulation of the composition thus presents an intriguing possibility for controlling memory stability by shifting the balance between labile and consolidated memories (Fig. 5 *E*–*G*). Therefore, this insight may pave the way to explore strategies to acquire resistance to memory loss.

## Materials and Methods

### *Drosophila* Husbandry.

Flies were raised on standard cornmeal food at 25 °C under a 12:12 h light–dark cycle (the light term starting at 8 AM) for all experiments. For the cell-type-specific knockout experiments, flies carrying GAL4 and UAS-Cas9 transgenes were crossed to flies with the guide-RNAs with ubiquitous U6 promoters, and F1 progenies were used. For cell-type-specific knockdown, flies with GAL4 were crossed with flies carrying UAS-conjugated double-stranded RNA (dsRNA), and F1 progenies were used. Flies aged 3 to 7 d posteclosion were used for experiments. All these manipulation experiments in this study employed constitutive downregulation of respective genes. As in-vivo lifetimes of synapse proteins can be long (97), constitutive and acute silencing could evoke different effects (27, 88).

The following *D. melanogaster* lines were used in this study: *GH146-GAL4* (BDSC #30026), *Synapsin-GFP_11_* (40), *Brp-GFP_11_* (40), *UAS-CD4::tdTomato* (BDSC #35841), *EYFP-Rab3* (58), *B2RT-STOP-B2RT-mCherry-Rab3, UAS-DSCP-B2R (BDSC #81505*, (98)) *UAS-Syt1::7xHalo7* (BDSC #67624), *R57C10-GAL4* (BDSC #39171), *R13F02-GAL4* (BDSC #48571), *UAS-uS-Cas9* (VDRC #340001), *UAS-uM-Cas9* (VDRC #340002), U6:3-*Rab3.gRNA* (BDSC #81906), *U6.2-Synj.dgRNA* (BDSC #92496), *UAS-Synapsin-dsRNA* (BDSC #82983). and *UAS-Rab3-GEF* (BDSC #78049).

### Generation of *Synapsin::GFP_11_*.

*Synapsin::GFP_11_* was generated by CRISPR/Cas9-mediated targeted integration as previously described (40). First, a 7xGFP_11_ cassette with a floxed 3xP3-RFP marker was inserted immediately in front of the stop codon of the RA isoform of the *Synapsin* gene. The 3xP3-RFP marker was subsequently removed by crossing the transformants to a *TM6B hs-Cre* balancer.

### Amnestic Treatment.

To induce cold anesthesia, flies were transferred to precooled empty vials and immersed in ice for 2 min. For isoflurane anesthesia, flies were placed in empty vials, and 10% (v/v) isoflurane (FUJIFILM Wako Pure Chemical Corporation, Osaka, Japan; catalog #095-06573) gas was introduced into the vials using a syringe. The flies were exposed to the gas for 2 min before being transferred to fresh empty vials. For CO_2_ anesthesia, flies were placed in vials filled with 100% CO_2_ gas for 2 min and subsequently transferred to empty vials. For concussion, flies were placed in empty vials and subjected to four consecutive mechanical impacts, resulting in a state of coma (99). This treatment caused no observable damage to the cuticle and legs of flies and killed less than 5% of flies, allowing the following behavioral characterization.

The recovery time was defined as the time between the offset of anesthetizing treatment and resumption of movement. To measure recovery time, anesthetized flies were immediately transferred to empty vials under a camera. Images were captured every 5 s, and flies were considered recovered at the first frame where a change in body position was observed compared to the previous frame.

### Olfactory Learning.

Standard differential olfactory conditioning with two odorants was used. Two odorants, 3-octanol (Merck) and 4-methylcyclohexanol (Sigma-Aldrich), were diluted to 10% in paraffin oil (Sigma-Aldrich) and used as conditioned stimuli (CS). Twelve pulses of 1.5 s 90 V electric shock were used as unconditioned stimulus (US). During training, flies were exposed to one odor (CS+) paired with the US, followed by another odor (CS−) presented without the US. Each step lasted 1 min, with 1-min intervals in between. In the test, flies were allowed to choose between the two odors in a T-maze for 2 min. Learning index (LI) was calculated using the formula: LI = (N_CS−_−N_CS+_)/(N_CS−_+N_CS+_), where N_CS+_ and N_CS-_ represent the number of flies in the arms with CS+ and CS− odors in the test, respectively. To account for any learning-independent odor preferences, a LI was calculated as the average across two reciprocally trained groups.

To evaluate the effects of amnestic treatments on 3-h memory (Fig. 1 *B* and *C*), flies were subjected to isoflurane anesthesia, CO_2_ anesthesia, concussion, or left untreated 1 h after training. Following these treatments, the flies were transferred to empty vials and allowed to recover at room temperature. Cold anesthesia was subsequently applied 2 h after training to assess whether it further impairs memory. After that, flies were allowed to recover for 1 h until tests in vials at room temperature. To measure 2-h ARM and total memory, which includes both ARM and ASM, flies were tested with or without cold anesthesia at 1 h after training as described above.

### Avoidance Tests.

To measure the responsiveness to odors and electric shock, avoidance of flies against these stimuli was tested in the T-maze. To test the electric shock responsiveness, flies choose electrified (12 pulses, 90 V) or nonelectrified arm in 1 min. For odor responsiveness, flies were given 2 min to choose between the two arms of the T-maze, one with the odor and the other one with paraffin oil. From the number of flies in the arms with (N_+_) and without (N_−_) shock or odor, avoidance index was calculated as follows: Avoidance index = (N_−_−N_+_)/(N_−_+N_+_). A positive avoidance index indicates avoidance from shock or odor.

### Brain Dissection and Immunohistochemistry.

To compare anesthetized and nonanesthetized flies, two methods of dissections were performed. For dissections without anesthesia, flies were placed in a 200 µL pipette tip with only the head exposed through the tip’s opening, and the heads were carefully separated from the bodies using tweezers. For dissections under anesthesia, flies were anesthetized as described above and transferred to a microscope stage while still anesthetized for dissection. All fly brains were dissected in ice-cold PBS. After dissection, brains were kept in ice-cold PBS with 2% paraformaldehyde for up to 30 min. For fixation, brains were incubated in 2% PFA in PBS for 1 h at room temperature. Fixed brains were washed in PBS containing 0.3% Triton X-100 (PBST) for 10 min 3 times and mounted in SeeDB2S (100) for confocal imaging.

For chemical tagging reaction, brains were incubated in PBST containing labeling substrates for 15 min at room temperature. Brains were washed in PBST for 10 min 3 times. The following labeling substrate was used at the indicated final concentration: Janelia Fluor® 549 HaloTag® Ligand (Promega, Madison, WI, USA; #GA1110; 500 nM).

For immunohistochemistry, fixed brains were incubated in 3% normal goat serum (NGS) for 30 min at room temperature for blocking. Brains were incubated in primary and secondary antibodies diluted in PBT with 1% NGS over two nights at 4 °C, respectively. After each step, brains were washed three times in PBT for longer than 20 min at room temperature and mounted in SeeDB2.

The following primary antibodies were used at the indicated concentrations: mouse monoclonal anti-Bruchpilot (nc82; Developmental Studies Hybridoma Bank, Iowa City, IA) at 1:100, mouse monoclonal anti-synapsin (3C11; DSHB) at 1:400, and rabbit polyclonal anti-GFP (Invitrogen, Waltham, MA, USA; #A11122) at 1:1,000. The following secondary antibodies were used at 1:200: Alexa Fluor 405 goat anti-mouse (#A31553; Invitrogen), Alexa Fluor 488 goat anti-mouse (#A11001; Invitrogen), Alexa Fluor 568 goat anti-mouse (#A11003; Invitrogen), and Alexa Fluor 488 goat anti-rabbit (#A11034; Invitrogen).

### Fluorescent Imaging of Adult Fly Brains.

For confocal imaging, brains were scanned with the Olympus FV1200 confocal microscope with following objective lens; 20× oil (NA = 0.85, UPLSAPO20XO, Olympus), 60× oil (NA = 1.42, PLAPON60XO, Olympus). The same scan settings were used for all samples in each experiment.

### Image Analysis.

All image analysis was conducted on Fiji (http://fiji.sc; RRID: SCR_002285). Confocal stacks were deconvolved by Richardson-Lucy methods. The theoretical point spread functions were used for deconvolution. For quantitative analysis in the presynaptic bouton of PNs, regions of interest (ROIs) were set manually based on genetically expressed markers such as CD4::tdTomato or Syt1::Halo7. For unbiased analysis, 10 to 20 boutons were randomly selected from the calyx without observing the measured signals.

To estimate the amount of fluorescently tagged molecules, mean pixel intensities were measured in the ROIs (Figs. 2 *C* and *E* and 3*B*). Background noise signals were removed by subtracting the mean signal intensities in the medium from the measured pixel intensities. In Fig. 3*E*, to remove background changes that are not specific to PN boutons, where *synj* was knocked out, the mean signal intensity in the protocerebral bridge of the same sample was subtracted from the measurement.

In Figs. 3 *G–I* and 4 *C*–*E*, nearest-neighbor distances between SV proteins and AZ were measured. Local intensity peaks were detected using the Find Maxima function in ImageJ. Peaks were identified as local maxima in each channel. Noise tolerance and minimum peak intensity were manually determined to allow detection of isolated bright spots. Same parameters were used for all compared datasets. For each local maxima of Brp signal, the nearest-neighbor peaks of SV proteins were measured.

In Fig. 5 *A*–*C*, Pearson’s correlation coefficient (R) was used to quantify the pixel intensity correlation between two-channel images. This was calculated using a custom macro in ImageJ. The formula for Pearson’s R is: R = Cov (Ch1, Ch2)/(SD_Ch1 * SD_Ch2), where Cov (Ch1, Ch2) represents the covariance between the two channels, and SD_Ch1 and SD_Ch2 are the SD of each respective channel. Calculation was done within each bouton using the manually set ROIs on single focal slices of deconvolved stack images. For this, the pixel size of all analyzed stack data was set to be 79 × 79 nm.

### Statistical Analysis.

Statistics were performed on GraphPad Prism5 (GraphPad Software; RRID: SCR_002798). Data were always first analyzed for normality (Shapiro–Wilk test) and for homoscedasticity (Spearman’s test or Bartlett’s test). If these assumptions were not violated, parametric tests (one-way ANOVA, followed by Dunnett’s test or multiple *t* test) were performed. If data failed to meet the assumptions for parametric tests, nonparametric tests (Kruskal–Wallis test, followed by Dunn’s post hoc pairwise test or multiple Mann–Whitney *U* test) were performed. When more than three groups were compared, *P*-values were adjusted using the Holm–Bonferroni method to control the family-wise error rate, or the Benjamini–Hochberg method to control the false discovery rate. For comparisons between two groups, the Mann–Whitney *U* test was used. For all figures, significance corresponds to the following symbols: n.s.: *P* > 0.05, *P < 0.05, **P < 0.01, ***P < 0.001, ****P < 0.0001.

## Supplementary Material

Appendix 01 (PDF)

## Data Availability

The datasets have been deposited in GIN (G- Node Infrastructure) services and can be accessed (https://doi.org/10.12751/g-node.rn7533) (101). All data generated or analyzed during this study are included in the manuscript and *SI Appendix*.
