# Supplementary material for: Disruption of a selective vesicle pool upon retrograde amnesia dissociates memory at presynaptic terminals
Source: Proc Natl Acad Sci U S A. 2026 Mar 5;123(10):e2514875123. doi: 10.1073/pnas.2514875123 (PMC12974456; doi:10.1073/pnas.2514875123)
Supplement: Supplementary file 1 — Appendix 01 (PDF) [file pnas.2514875123.sapp.pdf]

PNAS

Supporting Information for
Disruption of a Selective Vesicle Pool upon Retrograde Amnesia Dissociates Memory at Presynaptic Terminals

Shun Hiramatsu,[1]* Kaito Kabetani,[1] Shu Kondo,[2] Hiromu Tanimoto[1]*

[1] Graduate School of Life Sciences, Tohoku University, Japan.

[2] Department of Biological Science and Technology, Faculty of Advanced Engineering, Tokyo University of Science, Japan.

* Correspondence: Shun Hiramatsu (shunh_18@proton.me), Hiromu Tanimoto (hiromut@m.tohoku.ac.jp)

This PDF file includes:

Fig. S1 to S3

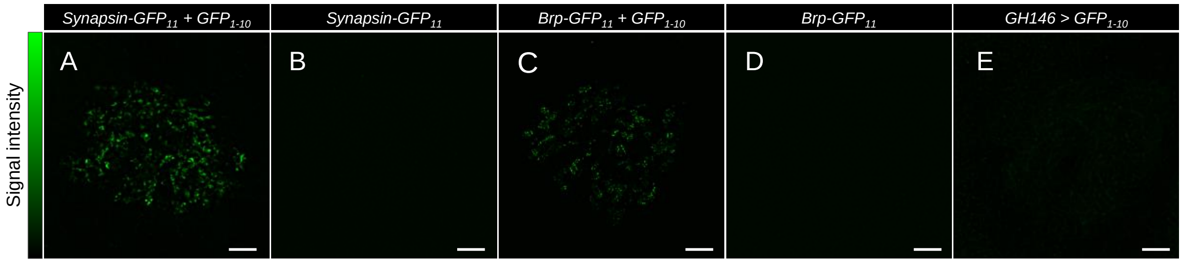

Fig. S1. Low background signals of $GFP_{1\text{-}10}$ and GFP_{11} without reconstitution.
(**A**, **C** and **E**) *GH146-Gal4* was used to express *UAS-$GFP_{1\text{-}10}$* in PNs. (**B** and **D**) Flies with *Synapsin::GFP_{11}* or *Brp::GFP_{11}* were dissected without *Gal4* and *UAS-$GFP_{1\text{-}10}$*. Single plane confocal images of the whole calyx are shown. To demonstrate low background signal, images from flies without GFP reconstitution (**B**, **D**, and **E**) were acquired using the same or higher detector gains than those for reconstituted GFP signals (**A** and **C**). Scale bars, 10 μm.

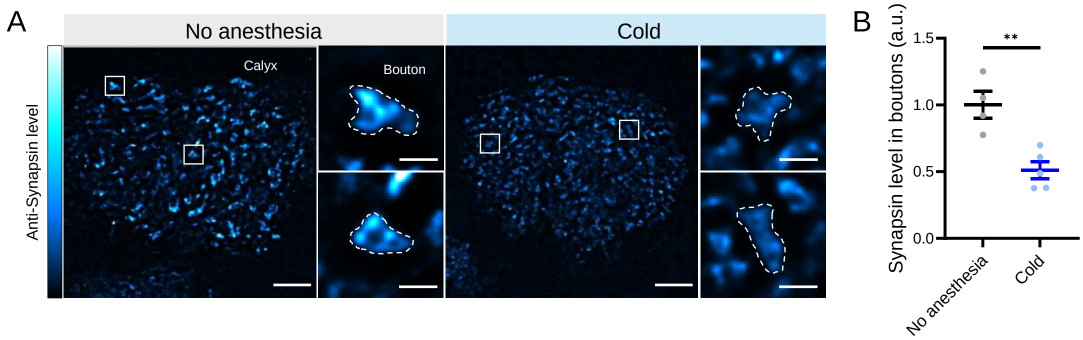

Fig. S2. An alternative dissection method reproduces the presynaptic reduction of Synapsin after cold anesthesia.

To rule out potential effects of stress on flies during trapping for dissection without anesthesia (Fig. 2A), flies were dissected without trapping. Instead, flies were immobilized in 70 % ethanol solution and dissected ("No anesthesia"). For comparison, cold-anesthetized flies are submerged in ethanol, and immediately dissected ("Cold"). Endogenous proteins were visualized by anti-Synapsin staining. (**A**) Single plane confocal images showing anti-Synapsin signals in PN boutons reproduced reduced Synapsin after cold anesthesia. Scale bars, 10 µm (low magnification) and 2 µm (high magnification). (**B**) The mean of 10 boutons from 4-5 calyx were measured to represent each brain sample. Mean ± SEM are shown. Unpaired t test was performed (N = 4-5; t = 4.276; p = 0.0037; **).

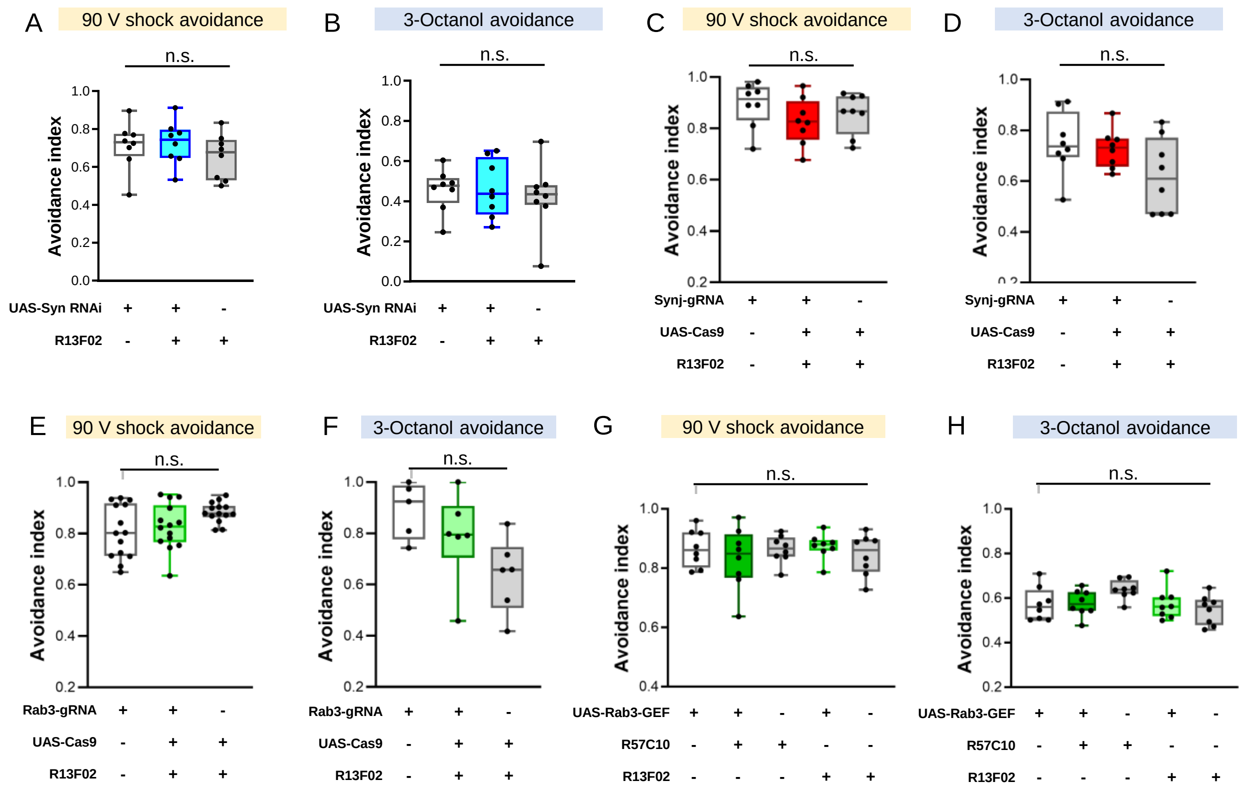

Fig. S3. Shock and odor sensitivity of flies used in learning experiment.

Avoidance against 90 V electric shock (**A, C, E and G**) and 3-octanol (**B, D, F and H**) were measured with flies used in learning experiment: *synapsin* knockdown (**A** and **B**; corresponding to Fig. 3A-3C), *synj* knockout (**C** and **D**; corresponding to Fig. 3K-3M), *rab3* knockout (**E** and **F**; corresponding to Fig. 4H-4J) and Rab3-GEF overexpression (**G** and **H**; corresponding to Fig. 5E-5G). Statistical summaries: (**A**): Kruskal-Wallis test showed no significant difference (N = 8; $H = 1.545$, $p = 0.4619$; n.s.). Mann-Whitney U tests with Holm-Bonferroni correction showed no significant differences: *CS* × *Synapsin-dsRNA* vs. *R13F02* × *Synapsin-dsRNA* ($p > 0.9999$; n.s.), *R13F02* × *Synapsin-dsRNA* vs. *R13F02* × *CS* ($p = 0.4866$; n.s.). (**B**): Kruskal-Wallis test showed no significant difference (N = 8; $H = 0.365$ $p = 0.8332$; n.s.). Mann-Whitney U tests with Holm-Bonferroni correction showed no significant differences: *CS* × *Synapsin-dsRNA* vs. *R13F02* × *Synapsin-dsRNA* ($p = 0.7237$; n.s.), *R13F02* × *Synapsin-dsRNA* vs. *R13F02* × *CS* ($p = 0.8045$; n.s.). (**C**): Kruskal-Wallis test showed no significant difference (N = 8; $H = 2.796$, $p = 0.2471$; n.s.). Mann-Whitney U tests with Holm-Bonferroni correction showed no significant differences: *Synj-gRNA* × *CS* vs. *Synj-gRNA* × *R13F02>Cas9* ($p = 0.193$; n.s.), *Synj-gRNA* × *R13F02>Cas9* vs. *CS* × *R13F02>Cas9* ($p = 0.5016$; n.s.). (**D**): Kruskal-Wallis test showed no significant difference (N = 8; $H = 3.436$, $p = 0.1794$; n.s.). Mann-Whitney U tests with Holm-Bonferroni correction showed no significant differences: *Synj-gRNA* × *CS* vs. *Synj-gRNA* × *R13F02>Cas9* ($p = 0.5958$; n.s.), *Synj-gRNA* × *R13F02>Cas9* vs. *CS* × *R13F02>Cas9* ($p = 0.406$; n.s.). (**E**): Kruskal-Wallis test showed a significant difference (N = 14; $H = 4.369$, $p = 0.1125$; n.s.). Mann-Whitney U tests with Holm-Bonferroni correction did not show significant differences: *Rab3-gRNA* × *yw* vs. *Rab3-gRNA* × *R13F02>Cas9* ($p = 0.7002$; n.s.), and *Rab3-gRNA* × *R13F02>Cas9* vs. *w* × *R13F02>Cas9* ($p = 0.1126$; n.s.). (**F**): Kruskal-Wallis test revealed significant difference among the three groups (N = 5-6; $H = 6.282$, $p = 0.0359$; n.s.). However, Mann-Whitney U tests with Holm-Bonferroni correction showed no significant differences: *Rab3-gRNA* × *yw* vs. *Rab3-gRNA* × *R13F02>Cas9* ($p = 0.3344$; n.s.), *Rab3-gRNA* × *R13F02>Cas9* vs. *w* × *R13F02>Cas9* ($p = 0.1157$; n.s.).

(**G**): Kruskal-Wallis test showed no significant difference (N = 8; H = 0.9768, p = 0.9133; n.s.). Dunn tests with Holm-Bonferroni correction showed no significant differences between *UAS-Rab3-GEF* × *CS* vs. *UAS-Rab3-GEF* × *R57C10* (p > 0.9999; n.s.), *UAS-Rab3-GEF* × *R57C10* vs. *CS* × *R57C10* (p > 0.9999; n.s.), *UAS-Rab3-GEF* × *CS* vs. *UAS-Rab3-GEF* × *R13F02* (p = 0.7646; n.s.), and *UAS-Rab3-GEF* × *R57C10* vs. *CS* × *R13F02* (p > 0.9999; n.s.). (**H**): Kruskal-Wallis test showed no significant difference (N = 8; H = 8.124, p = 0.0871; n.s.). Dunn tests with Holm-Bonferroni correction showed no significant differences: *UAS-Rab3-GEF* × *CS* vs. *UAS-Rab3-GEF* × *R57C10* (p > 0.9999; n.s.), *UAS-Rab3-GEF* × *R57C10* vs. *CS* × *R57C10* (p = 0.2636; n.s.), *UAS-Rab3-GEF* × *CS* vs. *UAS-Rab3-GEF* × *R13F02* (p = 0.9829; n.s.), and *UAS-Rab3-GEF* × *R57C10* vs. *CS* × *R13F02* (p > 0.9999; n.s.).
